# Structural Health Monitoring Using Ultrasonic Guided-Waves and the Degree of Health Index

**DOI:** 10.3390/s21030993

**Published:** 2021-02-02

**Authors:** Sergio Cantero-Chinchilla, Gerardo Aranguren, José Manuel Royo, Manuel Chiachío, Josu Etxaniz, Andrea Calvo-Echenique

**Affiliations:** 1 Department of Mechanical Engineering, University of Bristol, Bristol BS8 1TR, UK; sergio.canterochinchilla@bristol.ac.uk; 2 Electronic Design Group, University of the Basque Country (UPV/EHU), 48013 Bilbao, Spain; josu.etxaniz@ehu.es; 3 Techonological Institute of Aragón (ITAINNOVA), María de Luna 8, 50018 Zaragoza, Spain; jmroyo@itainnova.es (J.M.R.); acalvo@itainnova.es (A.C.-E.); 4 Department of Structural Mechanics & Hydraulics Engineering, University of Granada, 18001 Granada, Spain; mchiachio@ugr.es; 5 Andalusian Research Institute of Data Science & Computational Intelligence, University of Granada, 18001 Granada, Spain

**Keywords:** structural health monitoring, ultrasonic guided-waves, fatigue damage detection, transmission beamforming, degree of health index

## Abstract

This paper proposes a new damage index named *degree of health* (DoH) to efficiently tackle structural damage monitoring in real-time. As a key contribution, the proposed index relies on a pattern matching methodology that measures the time-of-flight mismatch of sequential ultrasonic guided-wave measurements using fuzzy logic fundamentals. The ultrasonic signals are generated using the transmission beamforming technique with a phased-array of piezoelectric transducers. The acquisition is carried out by two phased-arrays to compare the influence of pulse-echo and pitch-catch modes in the damage assessment. The proposed monitoring approach is illustrated in a fatigue test of an aluminum sheet with an initial notch. As an additional novelty, the proposed pattern matching methodology uses the data stemming from the transmission beamforming technique for structural health monitoring. The results demonstrate the efficiency and robustness of the proposed framework in providing a qualitative and quantitative assessment for fatigue crack damage.

## 1. Introduction

Crack initiation and propagation are the main driving forces of damage in many engineering materials [1]. In the absence of corrective actions, for example, decreasing the load levels or performing corrective maintenance actions, a crack may propagate up to the catastrophic failure of a structural component.

A wide range of non-destructive testing (NDT) techniques have been proposed to support operation and maintenance decision making [2,3,4]. Visual inspection, radiography, or ultrasound [5,6] are some examples of these NDT techniques; these are regarded as highly reliable but also time consuming (requiring the interruption of service) and dependent on the structure [7]. Alternatively, structural health monitoring (SHM) techniques enable a continuous *on-board* monitoring of the structural health facilitating the condition-based maintenance of the structure [8]. Some SHM techniques are especially suited for structures made of conductive materials such as metals (e.g., eddy currents [9]). Others, however, allow a more generic application to structures made of any kind of isotropic or anisotropic materials. For instance, strain gauges and fiber Bragg gratings [10] are typically used to monitor strain deformation in structures. However, their area of coverage is relatively low, thus potentially missing small defects unless a dense network of sensors is used. The acoustic emission technique has shown efficiency in fatigue damage detection and localization [11], although it is known as a passive method that requires capturing the instant of the damage growth in order to measure damage-related information. Alternatively, ultrasonic guided-waves [12,13] overcome most of the referred drawbacks and can be efficiently used as a non-intrusive and on-board SHM technique [14], due to their ability to actively explore large areas with a relatively small attenuation [15]. These beneficial properties of ultrasonic guided-waves have attracted the attention of safety-critical industries such the aerospace over the last few decades [16,17]. The aforementioned SHM technique uses piezoelectric wafer active sensors (PWAS) [18] permanently attached to the structure to generate and acquire ultrasonic guided-waves [19,20], which have to be further processed to infer damage-related information.

Several signal processing and damage inference strategies can be applied to extract damage information out of the ultrasonic data. A number of them are based on some forms of inverse problems that infer relevant damage parameters from the comparison between experimental and computationally simulated ultrasonic data [21]. For example, the localization of damage using ultrasonic guided-waves has been addressed by several authors using uncertainty quantification frameworks and time-of-flight (ToF) models [22,23]. The quantification of damage has also been addressed using ultrasonic measurements in the context of a probabilistic Bayesian inverse problem in [24,25]. An alternative approach for ultrasound-based damage inference is the time-reversal method [26], which makes it possible to focus ultrasonic guided-waves towards a defect in the structure without the need for a baseline. This technique overcomes the limitations stemming from both the wave dispersion and the unknown material deformations by focusing the signal [26]. Most of these methods are baseline free, hence needing no comparison between undamaged and non-pristine states. However, the latter is at the cost of employing a significant amount of physically-grounded model evaluations, which bounds its applicability in real-life real-time engineering scenarios not only for its computational cost but also for its model and implementation complexity.

Alternatively, the use of baseline-based methods for damage detection might render the required efficiency for complex structures. These are typically model-free, and a straightforward comparison between signal features obtained in the pristine state and the subsequent damage states is the only information required. In this sense, a number of methods have been proposed in the literature. One such example is the reconstruction algorithm for probabilistic inspection of defects (RAPID) [27] and further variations of the same [28]. This method is able to localize defects by using signal difference coefficients of sensor pairs. Also, in [27], the authors proposed the use of signal correlation coefficients to detect and monitor damage evolution in an aerospace panel. Other examples for the reconstruction of damage using a baseline from an undamaged state are the embedded-ultrasonics structural radar [29] and the delay-and-sum imaging algorithm [30]. These techniques use the difference between signals (acquired using a receiver beamforming mode) in healthy and damaged conditions to obtain only the signatures stemming from the defect [31,32,33]. More recently, an unsupervised feature-extraction method for online damage detection was proposed in [34], whereby a subset of the ultrasonic signals carrying the majority of the energy content was used. The baseline approach was also used in [35], where the effectiveness of the shear-horizontal guided-wave mode in monitoring damage was investigated. Besides, the change in the ToF of the signal peaks due to the presence of debonding in a composite plate was demonstrated in [36]. This group of approaches enables the inference of a lower degree of damage-related information compared to the aforementioned inverse problems, and they approach real-world engineering scenarios with a higher efficiency and model simplicity. Nevertheless, they still require a significant computational effort, which poses an important limitation for on-board SHM applications. Therefore, there is still a need to provide computationally efficient post-processing methods of ultrasonic guided-waves for real-time damage assessment.

To overcome this limitation, this paper proposes the use of a novel pattern matching post-processing method for the detection and monitoring of fatigue damage in isotropic materials based on signal features stemming from each characteristic point (CP) of the signal. These points correspond to the peak amplitudes (maximum and minimum) of the acquired signal. The ToF of the peaks are chosen as the base of comparison to detect and monitor damage in a structure [37]. More specifically, a set of these points acquired in the undamaged state are further used to build a trapezoidal function similarly to a membership function or fuzzy set [38,39]. Additional measurements in non-pristine states are evaluated in the proposed function, whereby a degree of health (DoH) of the structure is provided as output. The feasibility and efficiency of the proposed algorithm (based on [40]) are demonstrated in a real fatigue test of an aluminum plate. The ultrasonic transmission beamforming technique with a linear phased-array of six PWAS is adopted to monitor (i.e., excite and receive ultrasonic guided-waves) the structure with an enhanced power every 1000 loading cycles at the same load level. Another symmetric phased-array (which only receives ultrasonic signals) is used to support the results obtained from the former one. The experimental results show that the proposed methodology is able to detect fatigue damage at an early stage (i.e., the crack onset) and to monitor the crack growth in an effective and efficient manner. Furthermore, the ease of implementation of this methodology makes it potentially applicable to industrial environments in order to perform real-time SHM.

The remainder of the paper is organized as follows—Section 2 describes the proposed post-processing method based on the comparison of the ToF of the signal peaks; Section 3 shows experimental setup as well as the results obtained from this method in the fatigue experiment; Section 4 discusses the potential impact of the proposed methodology on ultrasonic guided-wave based SHM; finally, Section 5 provides concluding remarks and suggests future works.

## 2. Methodology

**Figure 1:**
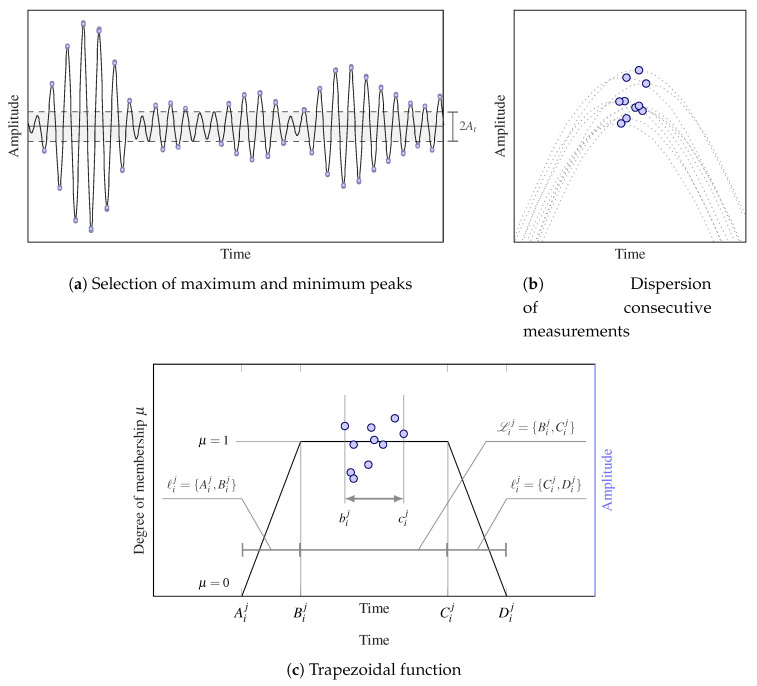
Panel (**a**): Selection of characteristic points (CPs) above the threshold value $A_{t}$. Panel (**b**): Illustration of ToF dispersion due to repeated measurements. Panel (**c**): Trapezoidal function used to evaluate the ToF mismatch based on the repeated measurements of one CP (blue circles).

The proposed detection and monitoring methodology based on a novel damage index is presented in this section. The index, referred to here as DoH, relies on ultrasonic guided-wave data taken from the SHM of structural panels. More specifically, the DoH uses baseline ultrasonic data acquired when the structure is in pristine state as a basis of comparison for further measurements when the structure is potentially damaged. To enhance the computational efficiency of this technique, only the ToF of the signal peaks above a user-specified amplitude threshold (named as $A_{t}$) are used as representative information of the raw ultrasonic data, as shown in Figure 1a. The peaks are obtained applying a sliding window to the ultrasonic signal. Note that the width of this window is chosen to be proportional to the period of the signal, that is, the inverse of the central frequency. The maximum and minimum points within these moving windows are selected, hence obtaining the signal peaks.

Furthermore, to partially address the irreducible uncertainty of these measurements and add robustness to the damage index, an arbitrary amount of repeated signals are acquired leading to a set of peaks with small differences in both time and amplitude (see Figure 1b). These variations are assumed to be an indicator of the measurement (or aleatory) uncertainty [41], which supports the previous robustness claim of the proposed damage index. Similarly to a fuzzy set, a trapezoidal function is created around the set of ToF points acquired in pristine state (see Figure 1c). This function makes it possible to assess the structural health by analyzing the ToF mismatch between the CPs (see Figure 1b for reference) acquired in both the degraded state and the pristine state. Note that as this function gives degree of membership values within the interval [0,1], the proposed damage index will carry qualitative information about the structural degradation.

Mathematically, the evaluation within the previously established trapezoidal function (or set) of CP${}_{i}^{j}$, namely the *i*-th CP of the *j*-th signal, i=1,…,N, j=1,…,m, will provide the degree of membership $\mu_{i}^{j}\in \left[0,1\right]$ of such a point to the set, which is defined as follows:

(1) $$\mu_{i}^{j}=\left\{\begin{array}{cc} 0 & {\mathrm{ToF}}_{i}^{j}\leq A_{i}^{j}\\ \frac{{\mathrm{ToF}}_{i}^{j}-A_{i}^{j}}{\ell_{i}^{j}} & A_{i}^{j}<{\mathrm{ToF}}_{i}^{j}<B_{i}^{j}\\ 1 & B_{i}^{j}\leq ToF_{i}^{j}\leq C_{i}^{j}\\ \frac{D_{i}^{j}-{\mathrm{ToF}}_{i}^{j}}{\ell_{i}^{j}} & C_{i}^{j}<{\mathrm{ToF}}_{i}^{j}<D_{i}^{j}\\ 0 & D_{i}^{j}\leq {\mathrm{ToF}}_{i}^{j}, \end{array}\right.$$

where ToF${}_{i}^{j}$ is the ToF of CP${}_{i}^{j}$ and $\ell_{i}^{j}=\left\{A_{i}^{j},B_{i}^{j}\right\}=\left\{C_{i}^{j},D_{i}^{j}\right\}$ is the ToF interval between $A_{i}^{j}$ and $B_{i}^{j}$, which results to be the same as the interval between $C_{i}^{j}$ and $D_{i}^{j}$. The proposed membership function as well as their related parameters are illustrated in Figure 1c. Note that the interval $L_{i}^{j}=\left\{B_{i}^{j},C_{i}^{j}\right\}$ is larger than the one obtained by measuring the dispersion of the CPs (i.e., $\left\{b_{i}^{j},c_{i}^{j}\right\}$) as shown in Figure 1c. This is to account for further measurement uncertainty that is not captured by the initial repeated measurements. Note also the intervals $\ell_{i}^{j}$ and $L_{i}^{j}$ need to be suitably defined by the modeler in the proposed approach.

Thus, when assessing damage through an acquired guided-wave, this method evaluates if CP${}_{i}^{j}$ falls within $L_{i}^{j}$, then $\mu_{i}^{j}=1$ meaning that the structure is unaltered. Alternatively, if the structure suffers a permanent damage, the ultrasonic guided-waves will be affected by this damage through slight ToF mismatches [42]. In this case, the CP${}_{i}^{j}$ is likely to fall within the interval $\ell_{i}^{j}$ due to an advance or delay of the acquired signal, which in turn leads to $\mu_{i}^{j}\in \left[0,1\right]$. Lastly, if CP${}_{i}^{j}$ falls out of the greater interval $\left\{A_{i}^{j},D_{i}^{j}\right\}$, then $\mu_{i}^{j}=0$ and it is assumed that the structure may have suffered a severe damage or significant modification.

The assessment of a degree of membership $\mu_{i}^{j}\in \left[0,1\right)$ may be an indication of structural damage, but it can also occur due to environmental or numerical noise. To overcome this issue, every CP${}_{i}^{j}$ is assessed and their degrees of membership $\mu_{i}^{j}$ are obtained, the global degree of membership of the *j*-th ultrasonic signal (i.e., M${}^{j}$, with M being the capital letter of μ) is obtained as follows:

(2) $$M^{j}=1-g\left({\left\{\mu_{i}^{j}\right\}}_{i=1}^{N}\right)=1-\min{\left\{\mu_{i}^{j}\right\}}_{i=1}^{N},\;j=1,\ldots ,m.$$

**Figure 2:**
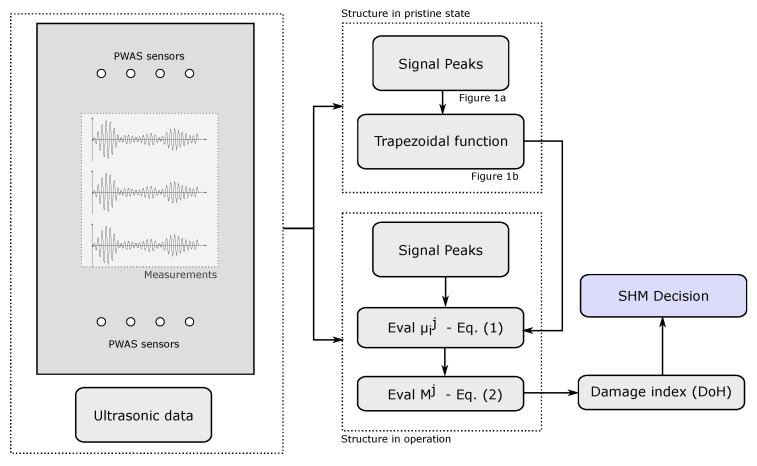
Schematic workflow of the methodology divided between the data acquisition of ultrasonic data and its post-processing depending on the actual structural state (i.e., pristine or in operation).

Note that the function g(·), conservatively chosen in this paper as the minimum of the degrees of membership, might be adopted differently such as the weighting and segmentation of the ToF or the amplitude [43]. Note also that in Equation (2) the function $g(\mu_{i}^{j})$ has been subtracted from the unity, consequently, M${}^{j}$ can be viewed as a damage index which increases as the defect becomes more severe, and vice versa. Moreover, when a set of ultrasonic signals are available and post-processed by this method, an array (or matrix) of M${}^{j}$ values rather than a single value can be obtained. The use of additional M${}^{j}$ values ultimately leads to a more reliable monitoring since the influence of sensor malfunctioning in the monitoring decision greatly decreases. In this case, the resulting array or matrix is referred here to as the DoH matrix of a structure, with values from 0 (maximum health) to 1 (maximum degradation). A schematic workflow of the proposed methodology is shown in Figure 2.

## 3. Case Study

The proposed DoH damage index (Section 2) for damage detection and monitoring is illustrated in this section. To this end, a fatigue test on an aluminum plate with a central notch has been carried out and its structural health is monitored by means of the DoH using ultrasonic guided-waves excited and received by PWAS and a SHM ultrasound system (SHMUS).

### 3.1. Fatigue Testing Configuration

**Figure 3:**
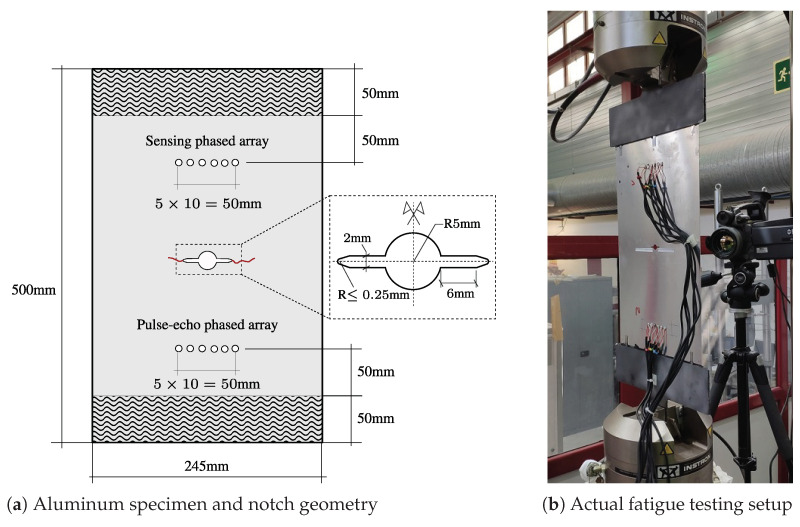
Panel (**a**): Schematic of the specimen and notch geometry, along with the position of the piezoelectric wafer active sensors (PWAS) arrays. Panel (**b**): Picture of M(T) aluminum specimen with two permanently attached phased-arrays mounted on the fatigue testing machine.

The fatigue test has been performed using a middle tension [M(T)] specimen with a centered crack and loaded in tension using a positive loading ratio. The test specimen of dimensions 245 mm × 500 mm × 1 mm has been extracted from a 1003x503 QQA250/5 ‘O’ 2024 aeronautic grade aluminum sheet using a water jet cutting procedure. The notch has been centered with respect to the test sample centerline and machined using a laser cutting procedure, with the final dimensions specified in Figure 3a. The total length of the machined notch is 22.5 mm approximately.

An Instron 8850 servo-hydraulic fatigue testing machine has been used to conduct the fatigue experiment. A fatigue pre-cracking procedure has been applied to develop a fresh and straight crack front to mitigate the effect of the machined started notch [44]. The crack onset and growth is recorded through a digital still camera. A tension-controlled fatigue test has been performed with a stress ratio ΔR=0.1 and maximum load amplitude $P_{max}=10$ kN, which corresponds to the 60% of the aluminum yield strength. The test was performed up to 100,000 cycles with a cycling frequency of 20 Hz. The reader is referred to Figure 3 for further information about the experimental set-up. Note that the upper and lower 50 mm bands of the specimen are used to clamp the plate into the fatigue testing machine.

### 3.2. Ultrasonic Guided-Wave Based Tests

**Figure 4:**
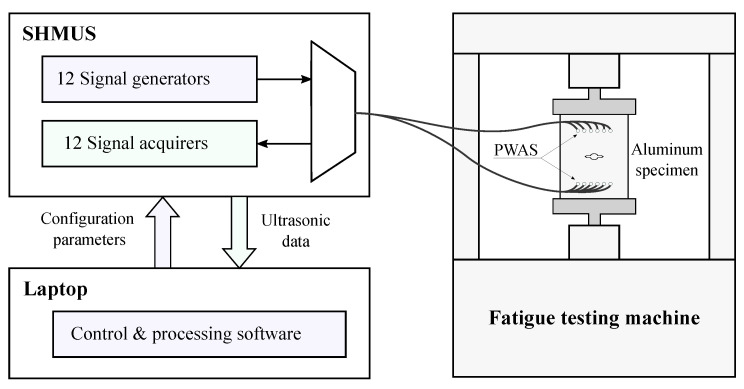
Schematic of the ultrasonic guided-wave based tests.

The detection and monitoring of the crack onset and growth has been carried out using two linear phased-arrays of six PWAS, consisting of six evenly spaced PWAS that are linearly placed in the structure [19]. Piezoelectric ceramic disc transducers of 7 mm diameter and 0.5 mm thickness have been used in this fatigue test. These are used as transmitter-receiver (T${}_{i}$) and receiver (S${}_{i}$) arrays for the generation and reception of the ultrasonic guided-waves, respectively. Note that the transmitter-receiver array (T${}_{i}$) works in pulse-echo mode by simultaneously generating and acquiring ultrasonic signals, while the receiver array (S${}_{i}$) functions as a sensor only, also known as pitch-catch mode. It is important to note that the PWAS T${}_{5}$ malfunctioned during the fatigue test and therefore its associated data have not been considered for SHM purposes. These PWAS have a radial mode of vibration and a resonant frequency centered at 300 kHz. They have been evenly spaced with a separation of 10 mm and symmetrically placed with regards to the sample centerline, as observed in Figure 3, and bonded to the aluminum surface using 3M™ Scotch-Weld™ DP490 epoxy adhesive. The piezoelectric transducers are managed by a SHMUS, which is a custom-built compact electronic device for ultrasonic guided-wave based SHM. It includes 12 arbitrary waveform generators and 12 acquisition systems (refer to [45] for further details of the SHMUS). The SHMUS is controlled by USB using a tailor-made control and processing software installed in a laptop. The schematic of the experimental set-up for damage monitoring is shown in Figure 4. The excitation signals are sinusoids of 4 cycles, with 300 kHz frequency (in accordance to the resonant frequency of the chosen PWAS) and 45 volts peak to peak amplitude. The ultrasonic signals are acquired using a sampling frequency of 60 MHz and 12 bits of resolution.

Furthermore, the transmission beamforming technique [46] has been adopted for the monitoring of the specimen during the fatigue test. Using this technique, synchronously delayed signals were applied to the T${}_{i}$ PWAS (refer to Figure 3a), so that the wave fronts can be constructively summed at a pre-established direction creating a main wave beam [47]. The ultrasonic tests have been carried out by steering the main beam at different directions, that is, from $\xi_{1}=0^{\circ }$ to $\xi_{37}=180^{\circ }$ with an increment of $\Delta \xi =5^{\circ }$, which sweeps the entire monitoring area. Note that the ultrasonic signals are acquired by both the T${}_{i}$ and S${}_{i}$ phased-arrays of PWAS, hence receiving two sets of 37 × 6 signals (i.e., 37 angles and 6 PWAS for each phased-array).

**Figure 5:**
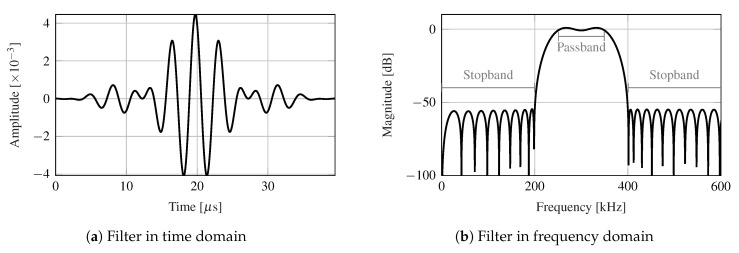
Time (panel (**a**)) and frequency (panel (**b**)) domain representations of the bandpass filter.

Programmed inspections during the fatigue experiment have been carried out every 1000 cycles. For each individual inspection, the fatigue test has been stopped at the minimum load level and an ultrasonic inspection has been performed using the transmission beamforming technique. The received ultrasonic signals (available in [48]) by the SHMUS have been de-noised using a bandpass filter centered at the frequency of excitation (i.e., 300 kHz). This allows a range of frequencies around the frequency of excitation to pass, while attenuating frequencies out of the scope. In this case the passband is defined in the interval [250 kHz, 350 kHz], while the stopbands, that is, attenuated frequency ranges, are defined within [0 kHz, 200 kHz] and [400 kHz, ∞) with a minimum attenuation of −60 dB. Figure 5 shows the representation of this bandpass filter in both time and frequency domains.

### 3.3. Damage Monitoring Results

The ultrasonic data acquired in pristine state at the minimum load level (i.e., 1.0 kN) have been used to build the trapezoidal functions (refer to Equation (1)). The parameters used to create these sets are: (1) amplitude threshold $A_{t}=15\%$ of the maximum amplitude of the signal in the time window considered for the analysis; (2) $\ell_{i}^{j}=10\%$ of the period of the excitation signal; and (3) $L_{i}^{j}=10\%$ of the same period in addition to the random dispersion measured from the 10 measurements. These values are selected so that measurements taken during the pristine state do not show any false damage indications. Moreover, $\ell_{i}^{j}$ and $L_{i}^{j}$ are set so that the measurement uncertainty is considered while avoiding that the trapezoidal functions of two adjacent peaks overlap. Note that the evaluation of a new signal in the previous functions has been carried out using a smaller amplitude threshold of $A_{t}=10\%$, so that a control loop is created for the amplitude in an analogous manner to a hysteresis controller [49].

**Figure 6:**
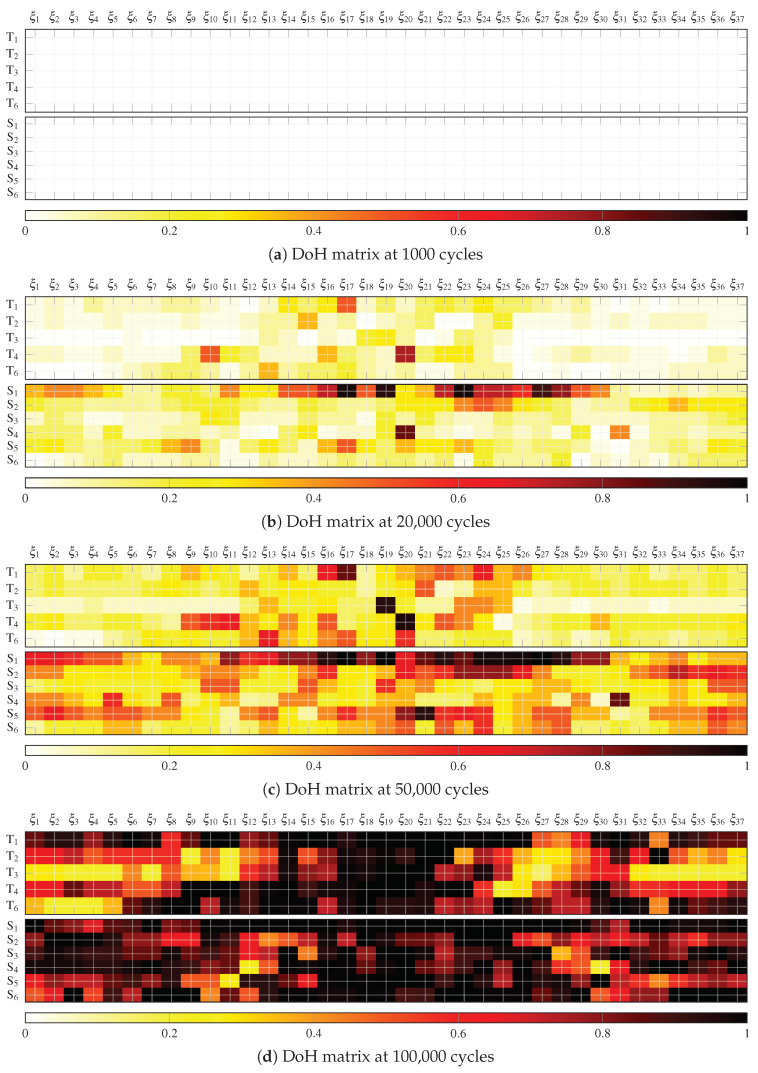
Degree of health (DoH) matrices at different fatigue cycles for both phased-arrays, that is, pulse-echo (T${}_{i}$) and pitch-catch (S${}_{i}$).

Figure 6a–d show the DoH matrix of the signals acquired at different fatigue cycles, namely 1000, 20,000, 50,000, and 100,000. Note that two DoH matrices are provided for each dataset, the one on the top is for the pulse-echo array (using T${}_{i}$ PWAS) and the one on the bottom is for the pitch-catch array (using S${}_{i}$ PWAS). As is evident from the results, a clear degradation of the structure is appreciated by the increase of the DoH damage index when increasing the number of cycles.

**Figure 7:**
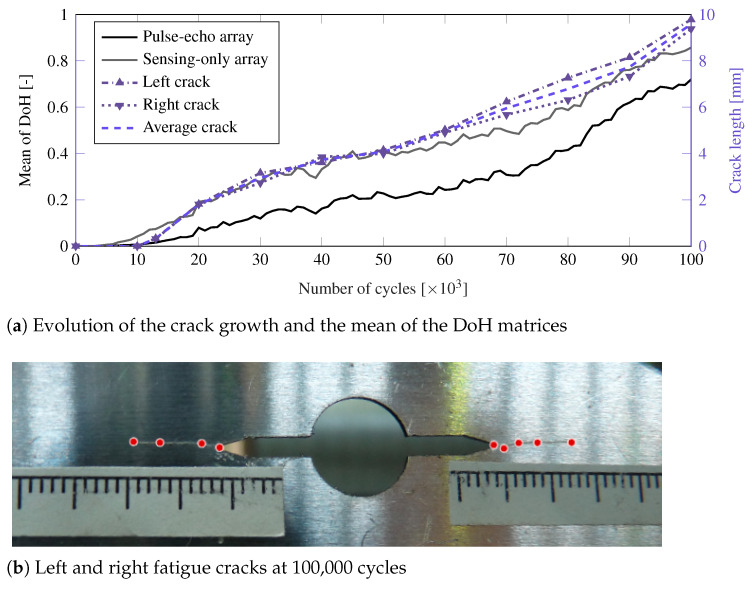
Evolution of the mean value of all DoH matrices obtained throughout the fatigue test in comparison with the crack length of the aluminum specimen in panel (**a**). Panel (**b**) shows the fatigue crack at 100,000 cycles along with the points used to digitize its length.

Notwithstanding, working with the DoH matrices may be limited in practice due to their complex interpretation. Hence the mean value of M${}^{j},\;\;j=1,\ldots ,m$ (i.e., all the values of the DoH matrices), is used here as a simplified statistic of the SHM data evolution, as shown in Figure 7a. Results also show that a higher damage index value is obtained using the data acquired in the S${}_{i}$ sensors (grey line) compared to the T${}_{i}$ sensors (black line). Furthermore, a comparison of the evolution of both the DoH index and fatigue crack length is also depicted in Figure 7a. The measurements of the crack length are obtained by digitizing different points in the crack path, as shown in Figure 7b. A remarkable agreement in the trend of both damage indicators is observed throughout the duration of the fatigue test. Note also that the early damage is detected using the two phased-arrays around 10,000 cycles, although the S${}_{i}$ sensors are able to provide an earlier indication of damage (i.e., even before it is visible by optical means).

**Figure 8:**
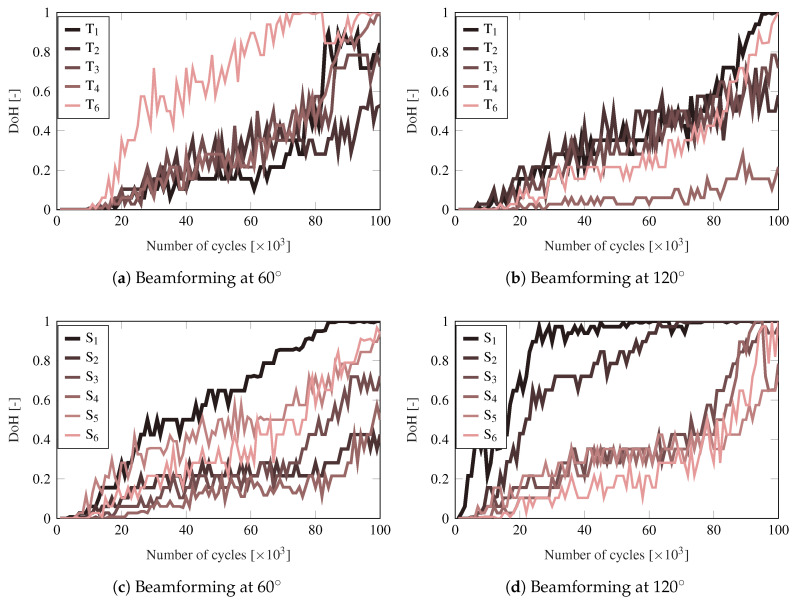
Evolution of M${}^{j}$ for the PWAS of the arrays in pulse-echo (panels (**a**,**b**)) and pitch-catch (panels (**c**,**d**)) modes at two particular directions.

Note that other statistics that illustrate the damage information obtained from the beamforming tests can be used. In particular, the adoption of the mean of the DoH matrix has shown accuracy in monitoring of the degradation of the structure. However, this statistic shows no indication of the dispersion of the cells of the DoH matrices and works as a filter of the evolution data. To provide such dispersion information, the evolution of the individual damage indices M${}^{j}$ for the array of sensors T${}_{i}$ when focusing at $60^{\circ }$ and $120^{\circ }$ are shown in Figure 8a,b, respectively. The equivalent data for the upper phased-array with S${}_{i}$ sensors are shown in Figure 8c,d. These curves depict the evolution of the $60^{\circ }$ column of the DoH matrices, equivalent to the ones shown in Figure 6.

As observed from Figure 8, the evolution of M${}^{j}$ for each PWAS has very different paths even for two adjacent PWAS. For instance, the M${}^{j}$ value of the sensors S${}_{2}$ and S${}_{3}$ at $120^{\circ }$ (see Figure 8d) have significantly different evolutions from 15,000 cycles onwards. This difference highlights the high sensitivity of the ultrasonic guided-waves when interacting with damage. Given that a different angle is formed between the damage and sensor, the reflected part of the incident wave may vary drastically [50]. Therefore, different sensor locations can acquire very different amplitudes of the scattered wave. Nevertheless, the damage onset is accurately captured by most of the sensors of the same phased-array at the same time.

## 4. Discussion

### 4.1. On the Ultrasonic Guided-Waves Results

The proposed methodology for damage detection and monitoring based on the DoH index, has been illustrated using ultrasonic guided-waves and a fatigue test. The damage index is based on pattern matching using fuzzy logic fundamentals, which provide it with robustness against measurement uncertainties. The adoption of the transmission beamforming technique for SHM entails the collection of large amount of data [46,51]. This allows a more robust damage detection, providing redundancy and ensuring an accurate monitoring in the event of hardware failure, for example, sensor malfunction. One basic assumption considered in this work is that the damage severity increases as does the ToF mismatch between signals acquired at different instants of time. Note that an important source of signal variability, which stems from environmental factors (e.g., temperature or humidity [52]), is not considered in this work. Additionally, changes in the properties of the coupling material between the PWAS and the structure will add variability on the received signals that is unrelated to the structural integrity. The consideration of these factors would imply the adoption of compensation techniques [53] and signal shape and amplitude prediction methods [54] so that the evaluation of the structural integrity becomes independent from these variables. This constitutes a desirable extension of the proposed methodology in order to monitor structures in the long term.

The ultrasonic tests were designed so that the wave beams focused at 37 directions using two phased-arrays: (1) a sensing array (S${}_{i}$) placed at the upper part of the plate in pitch-catch mode and (2) a generating one (T${}_{i}$), which was also acquiring in pulse-echo mode, placed at the bottom. In the literature, authors have typically used sensing PWAS in the opposite side of the damage [55] to measure ultrasonic guided-waves with higher amplitude and reduce the electronic complexities stemming from pulse-echo. To study their SHM performance, a comparison of both pulse-echo and pitch-catch approaches has been provided in this paper. In this context, Figure 7a shows that both S${}_{i}$ and T${}_{i}$ sensors were able to provide an accurate damage monitoring, although the sensors in the upper part of the plate (S${}_{i}$) showed a higher sensitivity for the early stages of damage. Notwithstanding this, the use of a pulse-echo approach is more practical in real-world engineering scenarios where operation and maintenance impositions may dictate the location of sensors and actuators.

The DoH matrices contain a large amount of damage-related information, which may be complex to interpret in practice. In this paper, we choose the mean value as a representative statistic of this large amount of SHM information, although other descriptive statistics could be adopted. This statistical simplification along with the computational efficiency of the signal post-processing, make the proposed methodology suitable for real-time and on-board SHM applications. Moreover, even in the event of a malfunctioning PWAS, the amount of post-processed signals is sufficiently large so that the mean of the DoH is capable of monitoring damage in an accurate manner (as shown in the results), further emphasizing the robustness of the proposed approach.

**Figure 9:**
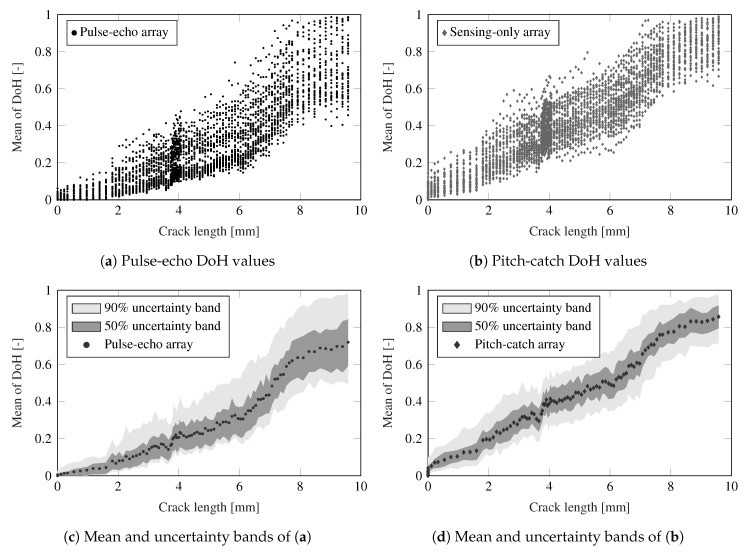
Relationship between crack growth and mean of DoH for both pulse-echo and pitch-catch working modes. Panels (**a**,**b**) provide the data for the 37 directions with an average value of DoH with respect to the PWAS in the array. Additionally, (**c**,**d**) show the mean and 90% and 50% uncertainty bands of the DoH data.

It is also noticeable from the results that a similar evolution pattern is obtained for both the proposed DoH index and the crack growth (refer to Figure 7a). To further investigate this relationship, both DoH indexes and their corresponding crack growth data are displayed in Figure 9. In particular, the data from the DoH matrices for each of the 37 directions, averaged over the six PWAS of each phased-array, are shown in Figure 9a,b, respectively, as compared to the measured fatigue crack length. These scatter plots reveal the dispersion of the DoH values with respect to the different monitoring directions used in the transmission beamforming test. However, a clear correlation is observed from these plots, and further revealed in Figure 9c,d which relate the mean of the DoH index and the crack length. The variability stemming from the 37 beamforming directions is also depicted using the 90% and 50% confidence bands. Note that although the array of S${}_{i}$ sensors provides a more certain index (with narrower bands), the T${}_{i}$ sensors in pulse-echo are still able to predict the crack length with a remarkable precision. This remarkable correlation suggest that the DoH index can be used to predict the crack length in a probabilistic manner. Furthermore, a state-space dynamical system representation of the data will provide a predictive model of the crack length whereby the remaining useful life of the structure can be estimated (see [56]), using just the DoH as input data. This further development would require the assessment of a higher number of specimens, which would provide additional data to build a robust predictive model.

### 4.2. On the Fatigue Properties of the Specimen

**Figure 10:**
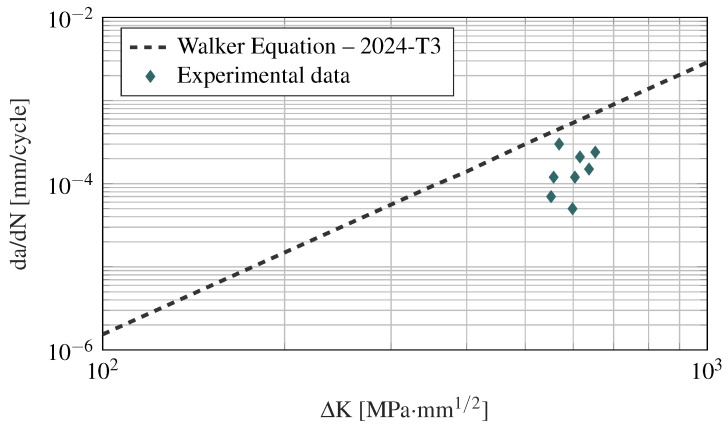
Crack growth data for QQ-A-250/5 ‘O’ M(T) aluminum specimen correlated with the stress intensity range.

The experimental results obtained in the aluminum M(T) specimen can be also assessed in terms of crack length evolution and rate for the adopted stress range and load ratio. It is worth mentioning that no appreciable change in stiffness was measured in the damaged specimen. The stress intensity range ΔK is obtained according to the indications of ASTM E647 for a M(T) specimen as follows [44]:

(3) $$\Delta K=\frac{\Delta P}{B}\sqrt{\frac{\pi \alpha }{2W}\sec\frac{\pi \alpha }{2}},$$

where α=2a/W, 2a is length of the machined notch in mm, *B* denotes the plate thickness in mm, and *W* is width of the specimen in mm. The parameter $\Delta P=P_{max}-P_{min}$ with $P_{max}$ and $P_{min}$ being the maximum and minimum load applied in the fatigue test, respectively. Notice that Equation (3) is valid for linear-elastic, isotropic, and homogeneous materials, and that it excludes potentially influencing effects derived from residual stresses or crack closure [57]. Note also that Equation (3) provides the values of ΔK with 1% accuracy for the clamped end specimen configuration used in the experimental fatigue test [44]. Additionally, the crack growth rate da/dN is obtained using the secant method, that is, calculating the slope of the straight line joining two adjacent points, as follows:

(4) $${\left(\frac{da}{dN}\right)}_{\bar{a}}=\frac{a_{n+1}-a_{n}}{N_{n+1}-N_{n}},$$

where $a_{n}$ and $N_{n}$ denote the crack length and the number of cycles of the *n*-th point, respectively. Given that the crack growth rate is an average over an increment (n+1), the average crack size $\bar{a}=1/2\left(a_{n+1}+a_{n}\right)$ is adopted to calculate ΔK [44]. Thus, the crack growth rate along with the stress intensity range are obtained from the experimental results and are shown in Figure 10. These data are compared to the Walker crack growth equation [58] adopting the parameters for a 2024-T3 clad and bare aluminum sheet, L-T (M2EA11AB1) [59]. It is noticeable from Figure 10 that the fatigue properties obtained from the experiments are relatively close to those available in the literature, considering the high variability that is typically manifested in fatigue tests [60]. This similarity is obtained even though (1) the fatigue tests do not strictly follow the ASTM E647 recommendations in terms of specimen aspect ratio; and (2) the data from the Walker equation is for an aluminum material with a different heat treatment than the one used in the tests.

Finally, a comment is added in relation to the correlation between crack length and DoH index. Indeed, motivated by the strong correlation of the proposed DoH index with respect to the crack length (as previously discussed in Section 4.1), an estimation of the stress intensity factor could also be provided as long as the crack growth rate was predicted. This estimation could be particularly useful for an efficient characterization of the structure under consideration, for example, to provide the stress state at a crack tip and to establish a fracture failure criterion [61]. In this sense, additional future lines of work are under consideration regarding data normalization and damage classification. At a mechanical test level, testing different sample geometries, materials such as carbon fiber reinforced polymers, and notch shapes are also required to validate the feasibility of the proposed damage index in monitoring and predicting damage features in more complex scenarios. However, it is worth mentioning that the proposed extensions for this DoH-based methodology related to the diagnosis of damage are valid for a controlled environment where only fatigue damage is carried out. Note that additional sources of damage, for example, impact or corrosion, would be accounted for in the DoH matrix, although the proposed descriptive statistic (mean value) would be unable to differentiate them. To overcome this limitation, pattern recognition approaches or machine learning could be applied to the DoH matrix data at the cost of a higher computational cost.

## 5. Conclusions

An ultrasonic guided-wave based post-processing approach for the detection and monitoring of damage in isotropic materials is proposed in this paper. The methodology relies on a damage index, named as the DoH index, which is based on the ToF variation of the characteristic points of signals. This index enables a real-time and computationally efficient continuous monitoring. The accuracy and robustness of the proposed method in assessing the health condition of a structure under fatigue load is illustrated using experimental data from a fatigue test in an aluminum M(T) plate. The following conclusions can be drawn:

- The proposed DoH damage index detects the damage onset and monitors its growth in a thin-walled metallic plate under fatigue testing conditions. It also shows a remarkable correlation with the fatigue crack length evolution.
- The damage evolution is accurately monitored using both the pulse-echo and sensing only phased-arrays of PWAS along with the transmission beamforming technique.
- The robustness of the proposed damage index builds on a pattern matching process, partially using fuzzy logic fundamentals, and information stemming from the transmission beamforming tests.
- The observed accuracy alongside the large amount of data contained in the DoH matrices encourage the use of the proposed method for a reliable diagnosis of damage features and mechanical properties.

Further work to improve and extend the proposed approach is under consideration regarding: (1) the application of environmental compensation techniques; (2) the embedment of the proposed damage index in a prognostics framework to predict the remaining useful life of the structure; and (3) the investigation of the damage index sensitivity with regards to different specimen geometries, materials such as composites, notch shapes, and damage forms along with their locations.

## Data Availability

All data used in this article are openly available for download from the University of Granada Research Data Repository at https://doi.org/10.30827/Digibug.65091.
